# The role of lipid oxidation pathway in reactive oxygen species-mediated cargo release from liposomes†

**DOI:** 10.1039/d4ma00535j

**Published:** 2024-10-12

**Authors:** Olga Lem, Roosa Kekki, Artturi Koivuniemi, Alexander Efimov, Timo Laaksonen, Nikita Durandin

**Affiliations:** a Tampere University, Engineering and Natural Science, Materials Science and Environmental Engineering Tampere Finland nikita.durandin@tuni.fi alexandre.efimov@tuni.fi; b University of Helsinki, Faculty of Pharmacy, Division of Pharmaceutical Biosciences Helsinki Finland timo.laaksonen@helsinki.fi

## Abstract

Reactive oxygen species (ROS)-mediated photooxidation is an efficient method for triggering a drug release from liposomes. In addition to the release of small molecules, it also allows the release of large macromolecules, making it a versatile tool for controlled drug delivery. However, the exact release mechanism of large macromolecules from ROS-sensitive liposomes is still unclear. There are no studies on the effect of lipid oxidation on the release of cargo molecules of different sizes. By using HPLC-HRMS method we analyzed the oxidation products of ROS-sensitive DOTAP lipid in phthalocyanine-loaded DOTAP:Cholesterol:DSPE-PEG liposomes after 630 nm light irradiation of different durations. Shorter illumination time (1–2 minutes) led to the formation of hydroperoxides and vic-alcohols predominantly. Longer 9-minute irradiation resulted already in aldehydes generation. Interestingly, the presence of epoxides/mono-hydroperoxides and vic-alcohols in a lipid bilayer ensured a high 90% release of small hydrophilic cargo molecules *i.e.* calcein, but not large (≥10 KDa) macromolecules. Oxidation till aldehydes was mandatory to deliver *e.g.* dextrans of 10–70 kDa with *ca.* 30% efficiency. Molecular dynamics simulations revealed that the formation of aldehydes is required to form pores or even fully disrupt the lipid membrane, while *e.g.* presence of hydroperoxides is enough to make the bilayer more permeable just for water and small molecules. This is an important finding that shed a light on the release mechanism of different cargo molecules from ROS-sensitive drug delivery systems.

## Abstract 2

Photooxidation of unsaturated lipids triggers cargo release from liposomes. Formation of hydroperoxides is sufficient for releasing small hydrophilic molecules, while aldehyde presence is mandatory for the release of larger macromolecules (>10 KDa).

## Graphical abstract

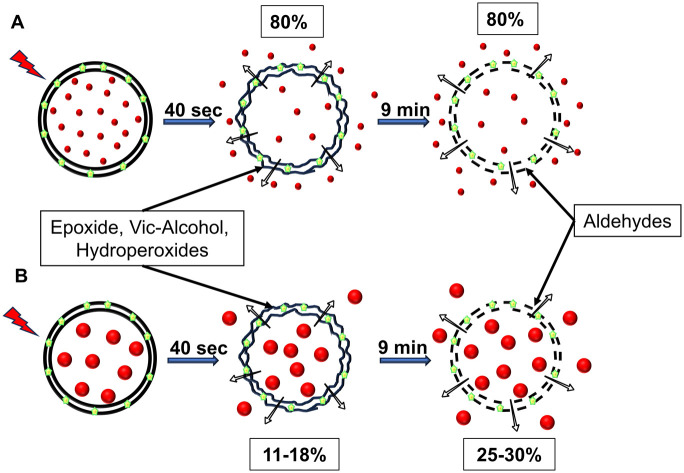

## Introduction

**Scheme 1:**
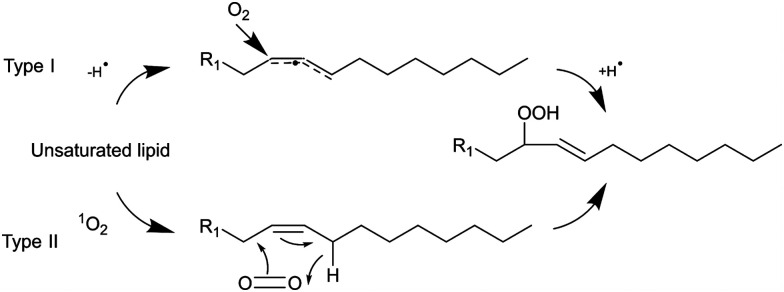
Type I and II reaction mechanisms to form lipid hydroperoxide.

Light can provide a non-invasive, selective, and safe trigger for releasing liposomal cargo through multiple activation pathways, such as photoisomerization, photocrosslinking, photocleavage, and light-induced oxidation *via* the generation of reactive oxygen species (ROS).^1–3^ Several studies have investigated membrane degradation through the photooxidation of phospholipids in ROS-sensitive liposomes, where different unsaturated lipids responsible for the ROS-sensitivity have been used, such as SOPC, SLPC, DOPC, DOPE, POPC, EPC, DLPC, and DOTAP.^4–9^ The common feature of all these lipids is the presence of carbon–carbon double bond. These can easily react with ROS, creating new lipid derivatives which can radically affect the structure and stability of lipid bilayers. The mechanism of lipids photooxidation has been investigated in several publications.^10,11^ It has been proposed that the mechanism can be classified as either type I or type II. The first type involves free radicals which are formed *via* interaction between a sensitizer in its triplet excited state and a substrate. The second type involves a singlet oxygen generated upon interaction of a sensitizer in the triplet exited state with a ground state oxygen.^12^ The radical chain reaction typically commences with the removal of an allylic hydrogen by a radical, leading to the formation of a carbon-centred radical. The oxygen then reacts with the formed radicals, resulting in peroxyl radicals (Scheme 1). Within this process, a hydrogen is abstracted from another lipid, producing a lipid hydroperoxide and a new lipid radical, or take alternative reaction routes that ultimately result in the formation of oxidized lipids with distinct organic functions such as aldehydes, alcohols, and ketones.^12^ Radical oxidation of lipids can yield both *trans* and *cis* isomers of hydroperoxides.^11^ The type II reaction is relatively simpler than the radical one since it generates fewer products. Particularly, energy transfer is happening from the triplet excited state of a sensitizer to a ground state oxygen, resulting in singlet excited state oxygen (^1^O_2_). Then the singlet oxygen reacts with an unsaturated bonds the lipids and directly produces hydroperoxides (Scheme 1).^12^

The oxidation of unsaturated lipids by singlet oxygen can increase the permeability of the lipid bilayer.^11,13–16^ For example, a combination of unsaturated lipids with a photosensitizer (PS), particularly DOPC/*m*-THPP has been shown to be an efficient system for the photo-triggerable release of small cargo molecules and a promising formulation in photodynamic therapy (PDT) and chemotherapy.^4^ The reported increase in lipid bilayer permeabilization was attributed to the formation of pores, as evidenced by various molecular simulations demonstrating the aggregation of phospholipid aldehydes leading to pore creation in the liposomal membrane after a certain irradiation time.^10,16,17^ Experiments with liposomes intentionally made of unsaturated lipids and lipid aldehydes confirmed an increased leakage of liposomes because of a lack of bilayer stability.^15,18,19^ However, the presence of different oxidation products in liposomal bilayer and their effect on the ROS-triggered release of different size cargo molecules has not yet been studied.

Liposomes, as hollow transporter structures for hydrophilic and hydrophobic drug compounds might provide a solution for the increasing of the treatment efficacy by combining PDT with chemotherapy. But in contrast to research on the controlled release of small molecules, there has been limited exploration of the triggered release of larger cargos like such as proteins and nucleic acids.^20,21^ This can be attributed to several challenges for the drug delivery systems: lower encapsulation efficiencies, the risk of protein aggregation, and difficulties in separating the released cargo from liposomes for analysis.^22^ The recent study showed the release of large macromolecules up to 500 kDa in size under red light illumination through ROS-mediated release.^23^ There, it was also suggested that the generation of oxidized lipids resulted in pore formation and thus accelerated the release of macromolecules. While there is a broad understanding of the overall processes involved in lipid oxidation, the specific chemical pathways through which photosensitizers and light induce permeability in lipid membranes are still not fully understood. Thus, the present study aims to assess the effect of pore formation on the release of large and small molecules from the phthalocyanine-loaded liposomes and the chemical steps during the photooxidation of lipids liposomal bilayer.

## Results and discussion

### Characterization of liposomes and release studies

**Fig. 1:**
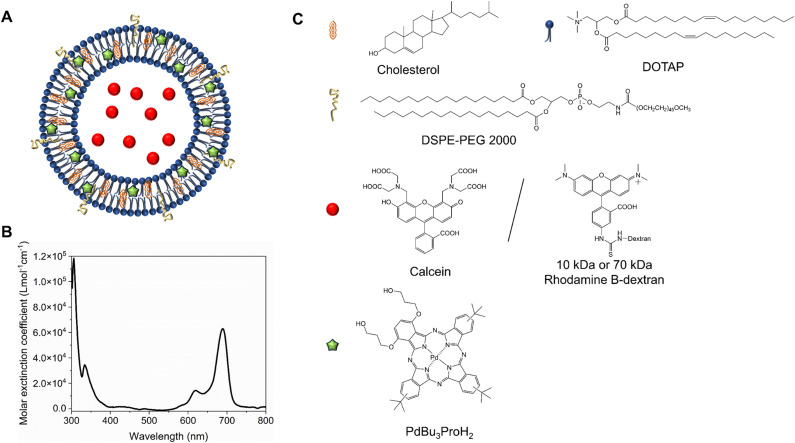
(A) Structure of liposome, with the following lipid composition DOTAP : Cholesterol : DSPE-PEG 2000 in ratio 45 : 50 : 5. (B) Absorption spectrum of PdBu_3_PrOH_2_ in Pyridine : H_2_O 3 : 1 (v/v). Chemical structure of cholesterol, DSPE-PEG 2000, Calcein, rhodamine B dextran, and PdBu_3_PrOH_2_.

**Fig. 2:**
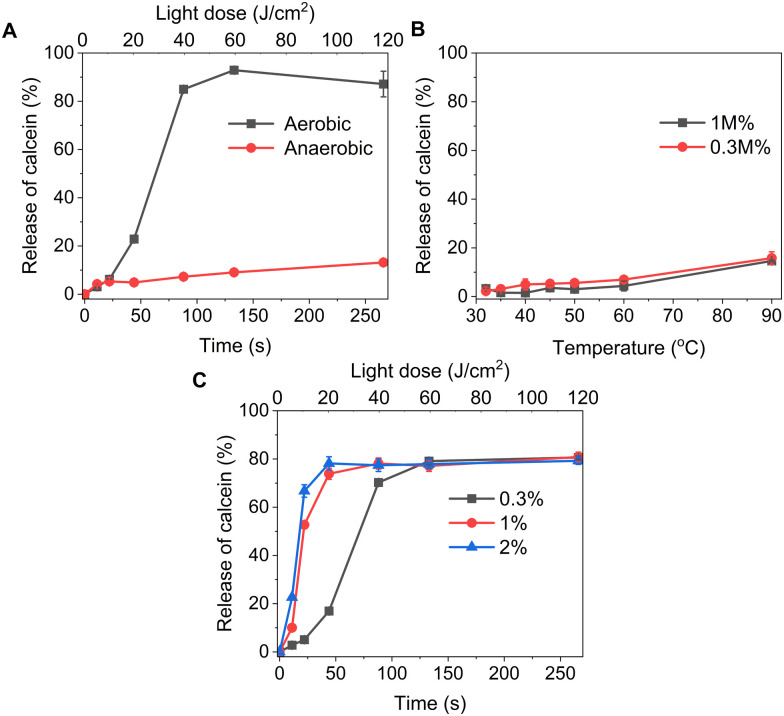
(A) Release of calcein from 0.3 M% PdBu_3_PrOH_2_ loaded liposomes at different illumination time under aerobic and anaerobic conditions. (B) Stability of liposomes 0.3 M%, and 1 M% PdBu_3_PrOH_2_ loaded liposomes at different temperatures. All experiments were done in 20 mM HEPES buffer (pH 7.4). (C) Release of calcein from 0.3 M%, 1 M%, and 2 M% PdBu_3_PrOH_2_ loaded liposomes under aerobic conditions at 37 °C.

We formulated light-sensitive liposomes with DOTAP as a major ROS-sensitive component (Fig. 1). Cholesterol was added to the formulation to increase the bilayer rigidity and phase transition temperature.^24^ The presence of DSPE-PEG can improve colloidal stability, avoid the formation of protein corona, and extend blood-circulation time.^25,26^ Hydrophilic cargo molecules covering a large range of molecular weights were encapsulated into liposomes, as shown schematically in Fig. 1A. Red/far-red absorbing sensitizer palladium(ii) phthalocyanine (PdBu_3_PrOH_2_) was inserted in the lipid bilayer. UV-visible absorption spectra of a novel palladium phthalocyanine was measured in pyridine : H_2_O 3 : 1 (v/v) and shown in Fig. 1B. The absorption maximum was observed at 690 nm, but for practical purposes, 630 nm wavelength laser was chosen to induce the cargo release from liposomes. As shown in Table 1, a series of liposomes with PdBu_3_PrOH_2_ (0.3–2 M%) were prepared that encapsulated either calcein or varying loadings of rhodamine B-labelled dextran (10 kDa and 70 kDa). Dextran-containing liposomes tended to be larger in size relative to the calcein-containing liposomes. This is because a different methodology was used in liposomes preparation for the large molecules: calcein-loaded liposomes were extruded with 100 nm pore membranes, whereas for the dextran-loaded liposomes, a simpler sonication process was used since the extrusion was unsuccessful for the larger cargos. Dextran-loaded liposomes also showed higher encapsulation efficiency, ranging from 4–10.5%, in comparison to *ca.* 2.6% for calcein-loaded liposomes. The encapsulation efficiencies of cargos are within a range expected for water-soluble molecules with no active loading mechanism. Furthermore, it has been observed that the encapsulation efficiency of the photosensitizer decreases as the mole ratio of the photosensitizer increases. We have determined that the loading efficiency of PdBu_3_PrOH_2_ grows with increasing molar ratio of the photosensitizer (Table S1, ESI†). All prepared liposomes showed a zeta potential higher than 21 mV, due to the presence of the positively charged DOTAP lipid. The thermal stability of calcein-loaded liposomes was also examined. These results presented exceptional stability of liposomes at temperatures up to 90 °C. The passive leakage did not exceed 20% release of calcein (Fig. 2B). A similar composition of liposomes was already published and showed good thermal stability due to the presence of cholesterol and DSPE-PEG 2000 in the lipid bilayer.^24,27^ The liposomes can therefore be considered very stable and will not release significant amounts of cargo molecules without light activation. Further, this suggests that the release must be related to photooxidation, and the possible photothermal contribution should be minor. The light-triggered release was performed with PdBu_3_PrOH_2_-loaded liposomes under various conditions. To determine whether the liposomes are ROS-sensitive, the light-triggered release was performed in 20 mM, pH 7.4 HEPES buffer under aerobic and anaerobic conditions (Fig. 2A). Illumination of 0.3 M% PdBu_3_PrOH_2_ liposomes for 266 seconds (120 J cm^−2^ light dose) was sufficient to induce at least 80% release of calcein in normal oxygen conditions. In contrast, only 15% of calcein was released in anoxic conditions using the same light exposure time. This demonstrates the oxygen-dependent nature of the release mechanism, indicating that the primary cause for cargo release is the generation of reactive oxygen species. Fig. 2C shows the effect of different PdBu_3_PrOH_2_ loadings on calcein release from the liposomes. 2 M% loaded liposomes showed the fastest release. In that case, irradiation for 22 seconds induced *ca.* 68% release of calcein, whereas the release from 0.3 M% and 1 M% PdBu_3_PrOH_2_ liposomes was only 8% and 55%, respectively. After a longer illumination time of up to 4 minutes, all PdBu_3_PrOH_2_-loaded liposomes reached the same maximum release of calcein. Thus, increasing the concentration of photosensitizer in the liposomal membrane did not increase the maximum release but accelerated the oxidation of the liposomal membrane *via* enhanced ROS generation (oxidation rates: *k* (2 M%) > *k* (1 M%) > *k* (0.3 M%) *i.e.* 2.265 > 1.784 > 0.389). Higher concentration of PS inside a bilayer leads to generation of higher concentration of reactive oxygen species that in turn accelerates the oxidation of unsaturated lipids in the bilayer.^28^ Thus, in our case, different concentrations of PS under similar conditions lead to an increased oxidation rate.

**Table 1:** Physicochemical properties of the prepared liposomes loaded with calcein and rhodamine B dextran (10 and 70 kDa)

PdBu_3_PrOH_2_, M%	Cargo	Size (nm)	PDI	Zeta potential (mV)	Encapsulation efficiency of cargo (%)
0.3	Calcein	140 ± 2	0.1	21.0	2.8 ± 0.1
1	Calcein	150 ± 1	0.1	25.8	2.6 ± 0.1
2	Calcein	150 ± 2	0.1	25.0	2.4 ± 0.4
0.3	10 kDa Dextran	309 ± 7	0.3	37.4	5.4 ± 0.1
1	10 kDa Dextran	527 ± 80	0.4	32.7	10.5 ± 3.9
2	10 kDa Dextran	488 ± 23	0.4	32.5	8.6 ± 4.3
0.3	70 kDa Dextran	667 ± 310	0.4	37.3	3.0 ± 0.9
1	70 kDa Dextran	490 ± 34	0.3	36.4	4 ± 0.0
2	70 kDa Dextran	481 ± 118	0.4	35.2	4.9 ± 0.7

**Fig. 3:**
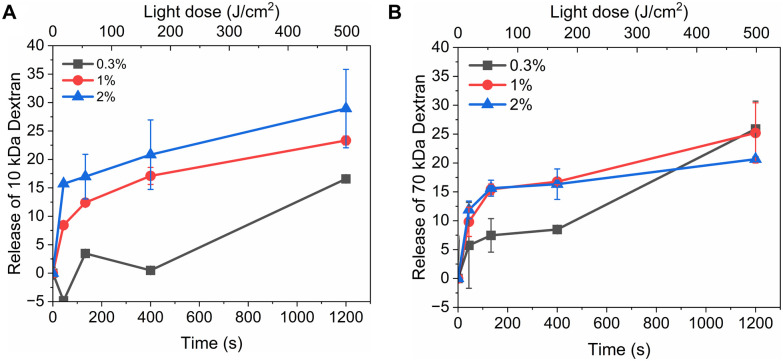
(A) Release of rhodamine B dextran 10 kDa from 0.3%, 1 M%, and 2 M% PdBu_3_PrOH_2_ loaded liposomes. (B) Release of rhodamine B dextran 70 kDa from 0.3%, 1 M%, and 2 M% PdBu_3_PrOH_2_ loaded liposomes. All experiments were done in 20 mM HEPES buffer (pH 7.4).

Fig. 3 shows light-triggered release from rhodamine B dextran-loaded liposomes. Three different loadings (0.3 M%, 1 M%, and 2 M%) of PdBu_3_PrOH_2_ and two different molecular weights (10 kDa and 70 kDa) were examined under the same experimental conditions. The maximum rhodamine B dextran release from PdBu_3_PrOH_2_ loaded liposomes was ranging from 14–29%. The maximum release % of dextran as well as release rate from liposomes (Table S2, ESI†) was increasing with higher loading of PdBu_3_PrOH_2_. This indicates that higher sensitizer loading leads to higher concentration of ROS produced. Indeed, the loading efficiency of the photosensitizer in liposomes containing 0.3 M% PdBu_3_PrOH_2_ was 0.38%, while in liposomes with 1 M% and 2 M% PdBu_3_PrOH_2_, it was 0.46% and 0.76%, respectively (Table S1, ESI†). Therefore, the total amount of the sensitizer was the highest in 2 M% sample in comparison to the other ones which led to a better release profile.

### Lipid oxidation and its reaction mechanism

**Fig. 4:**
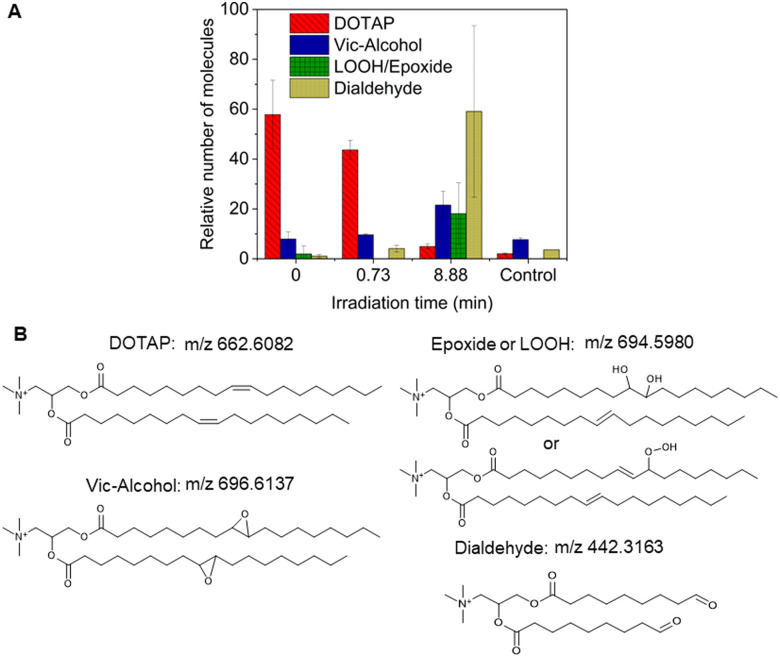
(A) Relative number of DOTAP and its oxidation products after different illumination times extracted from 0.3 M% PdBu_3_PrOH_2_ loaded liposomes (illuminated for 44 s and 9 min) and from deep oxidation control sample,1 M% PdBu_3_PrOH_2_ loaded liposomes (illuminated for 20 min). (B) Chemical structure of DOTAP and its oxidation products. Peaks of DOTAP and its oxidation products extracted from liposomal membrane were presented as molecules of lipids per 1000 molecules of DSPE-PEG.

**Fig. 5:**
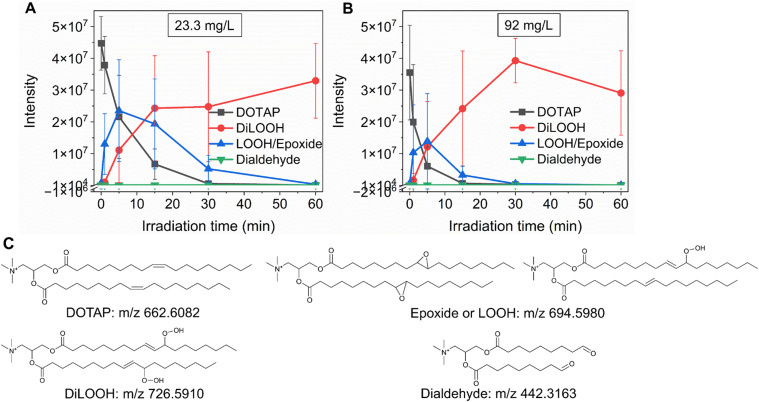
(A) DOTAP and its oxidation products in the presence of 23.3 mg L^−1^ (0.02 mM) PdBu_3_PrOH_2_ at different illumination times. (B) DOTAP and its oxidation products in the presence of 92 mg L^−1^ (0.1 mM) PdBu_3_PrOH_2_ at different illumination times. The initial concentration of DOTAP in both experiments was 3.14 g L^−1^ (4.7 mM). (C) Structures and molecular weights of DOTAP and its oxidation products. The photooxidation of DOTAP with various loadings of PdBu_3_PrOH_2_ was conducted in toluene.

**Fig. 6:**
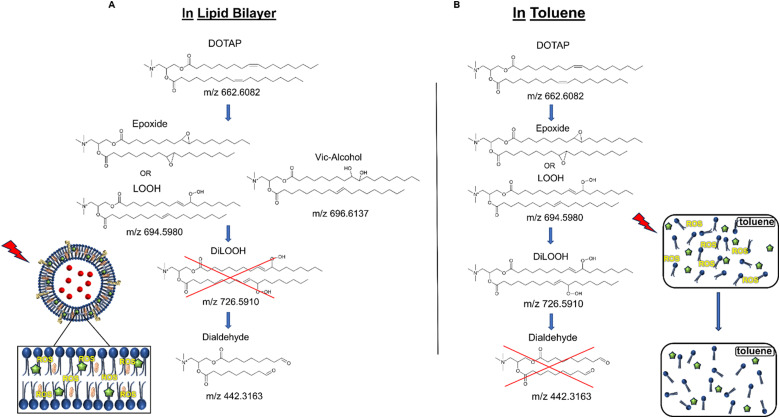
(A) DOTAP oxidation mechanism during illumination in liposomal membrane. (B) DOTAP oxidation mechanism during illumination in toluene.

**Fig. 7:**
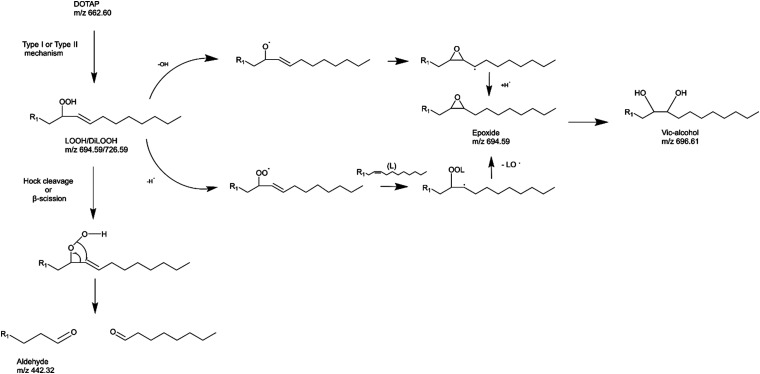
Possible reaction mechanism of DOTAP oxidation in liposomes.

As previously noted, the mechanism underlying the generation of lipid hydroperoxides through the oxidation of unsaturated lipids is well-established (Scheme 1). Nevertheless, the formation of further oxidation products remains a subject of debate, particularly within the context of liposomes oxidation. While there have been investigations addressing the influence of proximity on lipid oxidation products, the exact mechanism still requires further investigation.^7^ Thus, we have attempted to explain this concept through our own research findings. To determine the reaction mechanism of unsaturated lipid oxidation, we conducted HPLC-HRMS analysis on PdBu_3_PrOH_2_-loaded liposomes after different light exposures. As the DOTAP molecule is the most sensitive to singlet oxygen in the liposomal composition, our attention was directed towards the analysis of its oxidation products. To investigate lipid extracts, pre-established HPLC conditions were employed.^5,23^ The HPLC-HRMS analysis revealed the presence of different oxidation products of DOTAP, such as vicinal alcohol, epoxide, mono-hydroperoxide (LOOH), and aldehyde lipids (Fig. S1, ESI†). As shown in Fig. 4, the light exposure of the liposomes for up to 9 min resulted in a significant decrease in DOTAP concentration. The relative number of DOTAP molecules has decreased from 60 to almost 0. Simultaneously, this illumination led to a gradual increase in the quantity of oxidized lipids. After 44 seconds of illumination the maximum amount of vic-alcohols, epoxides/LOOH, dialdehydes was observed. Meanwhile, the quantity of DOTAP reached its minimum, implying that all DOTAP molecules had undergone oxidation. Following a 20-minute illumination period, both DOTAP and its oxidized products we were able to detect reached nearly zero levels, suggesting complete depletion. This phenomenon could be attributed to the potential further oxidation of aldehydes to carboxylic acids bearing lipids.^28^ Unfortunately, during HPLC-HRMS analysis, we encountered difficulties in detecting carboxyl-bearing lipids using both positive or negative electrospray ionizers, due to the zwitterionic nature of the molecules involved.^29^ It's worth noting that some of the observed oxidation products have been previously reported in various studies.^7,30^ However, a comprehensive understanding of the precise reaction mechanism within the liposomal bilayer requires further clarification. Therefore, we conducted additional experiments investigating DOTAP oxidation by PdBu_3_PrOH_2_ in toluene. In this case, the lipid is freely dissolved and does not form a bilayer. The system was illuminated for up to 1 hour in the presence of the photosensitizer (Fig. 5). As anticipated, after 60 min of illumination, a substantial quantity of hydroperoxides was detected in HPLC-HRMS. We were able to detect epoxides/mono-hydroperoxides (LOOH), and di-hydroperoxides (DiLOOH); however, no aldehydes were observed. Again, LOOH and epoxides have the same molecular weight and either/both molecules can be present. A distinct correlation was evident between the formation of epoxides and mono-hydroperoxides (LOOH), as well as di-hydroperoxides (DiLOOH), across varying illumination durations (Fig. 5). Following a 15-minute illumination period, the intensity of lipid epoxides or LOOH began to decline, while DiLOOH increased. Simultaneously, the intensity of DOTAP was barely noticeable, as illustrated in Fig. 5A. A similar trend was observed with a higher concentration of PdBu_3_PrOH_2_, although the changes occurred much more rapidly. The concentration of the photosensitizer in toluene is almost the same as in liposomal sample which is 41.4 ± 2.3 mg L^−1^ and 75.5 ± 14 mg L^−1^ for 0.3 M% and 1 M% PdBu_3_PrOH_2_-loaded liposomes, respectively (Table S1, ESI†). Therefore, through a comparative analysis of the oxidation products resulting from photooxidation in toluene *versus* those within the liposomal bilayer it becomes evident that oxidation in liposomes results in the formation of aldehydes, whereas in toluene, the process stops at di-hydroperoxides generation (Fig. 6). Considering these data alongside reported information, we propose a reaction mechanism for DOTAP oxidation in distinct environments (Fig. 7): the presence of hydroperoxides in both cases suggests that their formation may occur *via* either the type I or type II mechanism. However, in toluene, the reaction ends upon the formation of di-hydroperoxides. Given that no vicinal alcohol-bearing lipids were detected in toluene, it can be concluded that the reaction progresses through the formation of mono-hydroperoxide, which subsequently undergoes oxidation to form di-hydroperoxides. Noteworthy, the rate of DOTAP oxidation in toluene is significantly slower compared to that in liposomes. DOTAP was fully oxidized only after 30 minutes of illumination in toluene, whereas in liposomes, complete oxidation occurred within 20 minutes. Indeed, to achieve deeper oxidation of lipids into aldehyde formation, it is crucial that the distance between PS and the desired molecule should be no longer than 100 nm.^28^ With an average diffusion range of *ca.* 100 nm in water, ^1^O_2_ can only react with targets that are close to the PS although not necessarily in direct contact with it.^28^ Therefore, achieving the formation of aldehydes in toluene presents a challenge. The close proximity of the photosensitizer and lipids in liposomes allows for the reaction to proceed through different pathways. The presence of epoxides and vicinal alcohols products suggests that the reaction may proceed through hydrogen or hydroxyl radical abstraction, resulting in peroxyl or alkoxyl radical-bearing lipids. Following the abstraction of a hydroxyl radical group, the alkoxyl radical lipid may undergo cyclization to form an epoxide. Alternatively, the peroxyl radical lipid may dimerize with unsaturated lipid, subsequently yielding an epoxide, with the release of the second oxygen and its associated acyl chain as an alkoxyl radical. Additionally, epoxide formation can occur not only through radicals generation but also *via* catalysis by metal complexes, in this case, PdBu_3_PrOH_2_.^12,30^ Further oxidation of the epoxide through ring opening can result in the formation of vicinal alcohol.^31^ Regarding the photooxidation in toluene, the absence of epoxides or vicinal alcohols indicates that the oxidation of DOTAP predominantly proceeds through a type II mechanism involving the generation of singlet oxygen. Also, given the justification for the formation of epoxides and mono-hydroperoxides, we assert the presence of both species in liposomal membrane.

Simultaneously, hydroperoxide can undergo a transformation into aldehyde through hock cleavage or beta scission mechanisms. The hock cleavage mechanism involves a Lewis-acid-catalysed rearrangement of organic hydroperoxides, resulting in the oxidative cleavage of a C–C bond adjacent to the hydroperoxide group.^32^ The close proximity of PdBu_3_ProH_2_ and lipid molecules in the liposomal bilayer facilitates rapid oxidation of unsaturated lipids. The further hydroperoxide rearrangement also can be catalysed by the metal complex, leading to a subsequent formation of aldehydes.^30^ Due to the significantly accelerated oxidation rate in the liposomal bilayer compared to toluene, it is anticipated that certain intermediate products may be challenging or even impossible to detect. For instance, in our study, the observed concentrations of mono-hydroperoxides were consistently low. In summary, we propose that the oxidation process of DOTAP within the liposomal bilayer occurs through the generation of reactive oxygen species (ROS), catalyzed by PdBu_3_PrOH_2_.

### The role of lipid oxidation on cargo release from liposomes

**Fig. 8:**
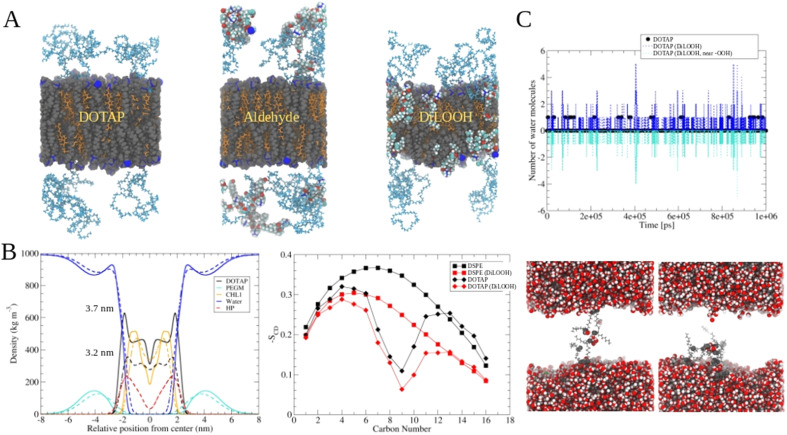
Molecular dynamics simulations of DOTAP-Cholesterol membranes with oxidized lipids. A Snapshots from the end of molecular dynamics simulations of DOTAP, aldehyde, and DiLOOH (di-hydroperoxides) systems. DOTAP and DSPE-PEG lipids are rendered with grey van der Waals spheres, cholesterol molecules are orange. Different atoms of aldehyde and hydroperoxide DOTAPs are colored according to the elemental type (red = oxygen, white = hydrogen and cyan = carbon). Blue spheres are nitrogen atoms. Water molecules were removed from the visualization for clarity. B (left) Density profiles and thicknesses for DOTAP and DiLOOH liposomal membranes (right) Lipid acyl chain order parameters for DOTAP and DSPE-PEG molecules in DOTAP and aldehyde simulations systems. C (top) Number of water molecules in the mid 1 nm of liposomal membranes of DOTAP and DiLOOH systems as a function of time (black and blue lines, respectively). Cyan line indicates the number of water molecules in contact with (within 0.5 nm) hydroperoxide oxygens as a function time. (bottom) DiLOOH system snapshots showing the water molecules at the center of membrane at simulation time points of 400 ns and 850 ns.

The release of the small hydrophilic molecule calcein from PdBu_3_PrOH_2_-loaded liposomes reached around 80% after *ca.* 88 s of illumination (Fig. 2A). Meanwhile, the oxidation of DOTAP had progressed to the formation of epoxides/mono-hydroperoxides and vicinal alcohols (Fig. 4). After the same illumination time, the release of 10 kDa and 70 kDa dextran was around 11–18% (Fig. 3). This implies that the presence of epoxides/mono-hydroperoxides and vicinal alcohols enhances membrane permeability by altering the hydrophobicity properties of the lipids which is enough for calcein to be released, but not for large dextrans.^10^ However, longer, 9–20 minutes illumination increased the release rhodamine B dextran to 15–30%. The further oxidation of mono-hydroperoxides progressed to the formation of aldehydes (Fig. 7). It has been hypothesized by several studies that the formation of aldehydes in liposomal bilayer promotes pore opening.^33^ This could have explained our results for the poor release of larger hydrophilic molecules which cannot easily permeate directly through lipid membranes containing epoxides/hydroperoxides. To shed the light on this phenomenon we conducted atomistic molecular dynamics simulations. Three different simulation systems were constructed for this purpose: 1. The intact liposomal membrane, referred to as the “DOTAP” system. 2. A liposomal membrane in which 33% of DOTAP molecules were oxidized to aldehydes, denoted as “aldehyde” (Fig. 5). 3. A liposomal membrane with 33% of DOTAP oxidized to di-hydroperoxides, denoted as “DiLOOH” (Fig. 5). During the simulations, both the DOTAP and DiLOOH systems remained integrated. However, in the case of the “Aldehyde” sample, the oxidation products of DOTAP were expelled from the membrane into the water phase, indicating that liposomal membranes with aldehyde oxidation products were not stable (Fig. 8A). Subsequently, we analysed the density profiles of “DOTAP” and “DiLOOH” systems. “Aldehyde” was not evaluated as all oxidation products were released from the membrane. In-depth analysis revealed that hydroperoxides render liposomal membranes thinner (Fig. 8B). We also found out that the presence of hydroperoxides increased the fluidity of the liposomal bilayer of the “DiLOOH” system (Fig. 8B). In addition, hydroperoxides increased the diffusion of water through the membrane, as the number of water molecules inside a one-nanometer slab is consistently higher compared to the non-oxidized membrane (Fig. 8C). Further analysis indicated that water molecules are predominantly localized next to hydroperoxide moieties within a one-nanometer slab of the membrane interior, suggesting that water molecules utilize hydroperoxide-rich areas for penetration (Fig. 8C). Therefore, our findings underscore distinct differences in the configuration of liposomal membranes based on the presence of lipid oxidation products. Particularly, hydroperoxides do not compromise membrane integrity yet facilitate water permeation through it. This elevated permeability already enables a release of relatively small hydrophilic molecules from the liposomes following the shorter exposure time (40 s–1 minute) studied in this work. Whilst, aldehyde oxidation products of DOTAP formed after a longer illumination period contribute to more pronounced instability of liposomal bilayer, ultimately leading to pore formation and/or collapse of liposomes. Hence, we propose that pore formation is one of the main contributing factors in the release of large 10–70 kDa macromolecules.

## Conclusion

The loading of large 10 and 70 kDa macromolecules and small calcein molecule into ROS-sensitive liposomes and their release upon red 630 nm light exposure was successfully achieved using PdBu_3_PrOH_2_ phthalocyanine-based hydrophobic sensitizer. The excitation of the sensitizer resulted in generation of ROS followed by the oxidation of an unsaturated membrane lipid, DOTAP. We demonstrated that shorter illumination times predominantly led to the formation of hydroperoxides, epoxides and vic-alcohols; whereas longer irradiation resulted in the formation of aldehydes. Our study showed that the presence of epoxides/mono-hydroperoxides and vic-alcohols facilitated the efficient release of ∼ 600 Da hydrophilic cargo molecules. The oxidation of lipids down to aldehydes which led to a formation of pores in the membrane was essential for the efficient release of 10–70 kDa macromolecules. A comparative study of lipids’ oxidation in the liposomal membrane and in toluene solution helped to better understand chemical pathways behind DOTAP oxidation. We concluded that in contact-dependent conditions when the sensitizer is localized next to the oxidizable lipid, the oxidation of DOTAP into aldehydes *via* the oxidative cleavage can occur due to the proximity of a photosensitizer to an unsaturated lipid. In contact-independent conditions in solution the reaction is limited to the oxidation into hydroperoxides.

## Experimental

### Materials

1,2-Dioleoyl-3-trimethylammonium-propane (DOTAP) and 1,2-distearoyl-*sn*-glycero-3-phosphoethanolamine-N [maleimide(polyethylene glycol)-2000] (DSPE-PEG) were purchased from Avanti Polar Lipids (USA). Cholesterol, HEPES buffer, chloroform, Triton X-100 (10% solution), calcein, sodium sulfite (Na_2_SO_3_), sodium hydroxide (NaOH), formic acid, and ammonium formate, sodium tetrachloropalladate(ii), dimethylformamide (DMF), methanol (MeOH), acetonitrile, silica 60, silica 100, dichloromethane (DCM), ethanol, 3,6-di(hydroxypropyloxy)phthalonitrile, 4-*tert*-butylphthalonitrile were purchased from Merck (Germany). The rhodamine B dextran was ordered from Thermo Fisher Scientific (USA). All chemicals and solvents were used as received.

#### Synthesis of 1,4-di[hydroxypropyloxy]-9(10),16(17),23(24)tri[*tert*-butyl]phthalocyanine (H_2_Bu_3_PrOH_2_)^34^

**Scheme 2:**
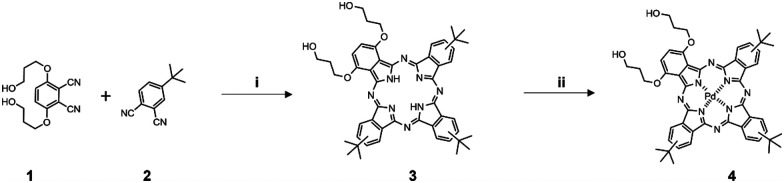
Synthetic route to PdBu_3_PrOH_2_ (4). Reagents and conditions: (i) Li, 1-pentanol, reflux, Ar, 2.5 h; (ii) sodium tetrachloropalladate(ii), DMF, reflux, argon, overnight.

1,4-Di[hydroxypropyloxy]-9(10),16(17),23(24)tri[*tert*-butyl]phthalocyanine (H_2_Bu_3_PrOH_2_) was synthesized by the same method as previously described. Briefly, free base phthalocyanine was synthesized by mixing 3,6-di(hydroxypropyloxy)phthalonitrile and 4-*tert*-butylphthalonitrile for 2.5 h under argon atmosphere at reflux. The collected product was purified by two-column chromatography on Silica 100, eluting the first fraction with CHCl_3_, and the second fraction with CHCl_3_ : EtOH 18 : 1 on both columns, consequently. The second fraction was purified on Silica 60 by using same eluting systems as before. Then collected product was crystallized by washing it with acetonitrile (Scheme 2). UV-vis: *λ*_max_(CHCl_3_/EtOH 1 : 1)/nm 340 (*ε*/dm^3^ mol^−1^ cm^−1^ 59 122), 693 (89 631), 719 (84 165). NMR: *δ*_H_(300 MHz; CDCl_3_/CD_3_OD 10 : 1; Me4Si) 9.50–8.45 (6H, m, phthalo-H), 8.28–8.07 (3H, m, phthalo-H), 6.88–6.07 (2H, m, 2,3-phthalo-H), 4.65–4.24 (8H, m, OCH_2_CH_2_CH_2_OH), 2.51–2.23 (4H, m, OCH_2_CH_2_CH_2_OH), 1.91–1.68 (27H, m, C(CH_3_)_3_), −2.40 to 2.97 (2H, m, NH), CH_2_OH were not resolved. MS (ESI-TOF; CHCl_3_/MeOH 1 : 1): *m*/*z* 831.4359 (M+H)^+^ (calcd for C_50_H_54_N_8_O_4_ 831.4346).

#### Synthesis of [1,4-di[hydroxypropyloxy]-9(10),16(17),23(24) tri[*tert*-butyl]phthalocyaninato (2-)-N29,N30,N31, N32]palladium(ii), PdBu_3_PrOH_2_

Sodium tetrachloropalladate(ii) (20 mg, 0.07 mmol) and 1,4-di[hydroxypropyloxy]-9(10),16(17),23(24)tri[*tert*-butyl]phthalocyanine (20 mg, 0.02 mmol) was dissolved in dimethylformamide (10 mL) and the reaction was heated at reflux overnight under argon atmosphere. The solution was cooled to room temperature, evaporated on the Rota-vap, and washed with dichloromethane (DCM). After washing the collected product was purified by column chromatography on Silica 60 eluting with DCM : MeOH 20 : 1. The second fraction was purified by preparative TLC eluting with DCM : MeOH 20 : 1. The collected product was obtained as a dark blue powder (10 mg, 55%) after drying (Scheme 2). UV-vis: *λ*_max_(pyridine : H_2_O 3 : 1)/nm 335 (*ε*/dm^3^ mol^−1^ cm^−1^ 31 390), 690 (62 852). NMR: *δ*_H_(500 MHz; CDCl_3_/CD_3_OD 10 : 1; Me4Si) 8.90–8.7 (6 H, m, phthalo-H), 7.90–7.7 (3 H, m, phthalo-H), 7.7–7.3 (2 H, m, 2,3-phthalo-H), 4.3–3.9 (8 H, m, OCH_2_CH_2_CH_2_OH), 2.2–2.0 (4 H, m, OCH_2_CH_2_CH_2_OH), 1.7–1.4 (27 H, m, C(CH_3_)_3_). MS: *m*/*z* 955.30562 ((M+Na)^+^) (calcd for C_50_H_54_N_8_O_4_Na 955.30453).

### Liposomes preparation

For the formulation of the liposomes, DOTAP, DSPE-PEG, and cholesterol were dissolved in chloroform, and mixed in a molar ratio of 45 : 5 : 50. After the addition of 0.3–2 mole % of PdBu_3_PrOH_2_ dissolved in chloroform, the mixture was placed in a rotavapor. The chloroform was evaporated for 60 minutes at 67 °C under nitrogen flow at low pressure, gradually reducing the pressure to 80 mbar. The thin lipid film was hydrated with 1 mL of calcein solution (30 mM, pH 7.4), or with 1 mL of 70 kDa rhodamine B dextran solution (0.1 mM in HEPES, pH 7.4), or with 10 kDa rhodamine B dextran solution (0.143 mM in HEPES, pH 7.4). The suspension was hydrated for 3–4 hours at 67 °C and frequently vortexed. The calcein-loaded liposomes were extruded 11 times through a polycarbonate porous membrane with a 100 nm pore size. The liposomes were purified by using size-exclusion chromatography (SEC) on Sephadex G-50 gel filtration medium, eluted with a HEPES buffer (pH 7.4).

The rhodamine B dextran loaded liposomes were sonicated for 20 min at 60 °C by using an ultrasonic water bath. 1 mL of the rhodamine B dextran-loaded liposomes was diluted with 30 mL of HEPES buffer and ultracentrifuged in 32 mL conical centrifuge tube for 1 h (100 000 g, 4 °C, at vacuum). After the ultracentrifugation, the supernatant was removed, and the liposomal pellet was resuspended with the appropriate amount of HEPES buffer.

### Characterization of the PdBu_3_PrOH_2_ liposomes

The diameter and zeta potential of the liposomes was measured with a dynamic light scattering (DLS) using Zetasizer Nano Series instrument, Malvern Instruments (United Kingdom). The liposome sizes are reported by *z*-average mean values. For the zeta potential measurements, a DTS1070 Zetasizer measurement cell was used.

### Calcein release from PdBu_3_PrOH_2_ liposomes

The purified PdBu_3_PrOH_2_-liposome sample were diluted with HEPES buffer (1 : 10, pH 7.4). The liposome samples were light-activated with 630 nm, 450 mW cm^−2^ Modulight ML8500 automatic biomedical illumination system (Modulight, Finland) for 11–266 seconds. A dark control sample in the same 96-well plate was shielded from light. To determine the maximum release from the PdBu_3_PrOH_2_ liposomes, 10 μL of 10% Triton-X was added to a control sample in another well of the 96-well plate. The fluorescence of released calcein was measured by a Varioskan Lux plate reader, Thermo Fisher Scientific (USA) at excitation and emission wavelengths of 493 nm and 518 nm respectively. Each study was conducted in triplicate, and the mean value and standard deviation were calculated. The release percentage of calcein was calculated by using eqn (1).1
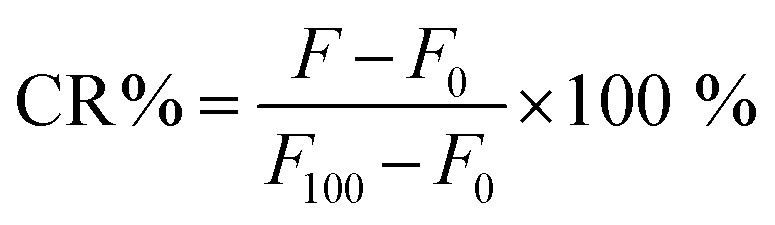


The *F* is the fluorescence of the sample at a specific measurement point, *F*_0_ is the background fluorescence of the cold control sample, and the *F*_100_ is the maximum release of calcein following the addition of 10% Triton-X. CR% is calcein release percentage. The rate constants of cargo released from the liposomes were extracted from the linear fitting equation by using Origin software. (See Fig. S3 and Table S2, ESI†)

### Stability studies of liposomes

The release of calcein was studied at temperatures ranging from 32 to 90 °C on a Eppendorf ThermoMixer® C (Eppendorf, Germany). The samples (10 μL) were added to HEPES buffer (490 μL) and heated for 10 min while shaking at 300 rpm, following a fluorescence analysis by using a Varioskan Lux plate reader (Thermo Fisher Scientific Inc., Waltham. MA, USA) as previously described. The maximum release of calcein was determined by the addition of 10 μL of 10% Triton-X and the background fluorescence was determined by analyzing liposomal samples kept at +4 °C temperature.

### Rhodamine B dextran release from PdBu_3_PrOH_2_ liposomes

The purified and diluted liposome samples (1 : 10 in HEPES buffer, pH 7.4) were pipetted to the well plate in quadruplicates. The liposome samples were light-activated with 630 nm, 450 mW cm^−2^ Modulight ML8500 illumination system from 44 seconds −20 min. A dark control sample in the 96-well plate was shielded from the illumination. After the light irradiation, the samples were ultracentrifuged to separate the released dextrans from the liposome-encapsulated dextran quantity by placing the pooled samples in an ultracentrifuge tube. The samples were ultracentrifuged for 1 h (100 000 g, 4 °C, at vacuum). After the ultracentrifugation, the supernatant was collected in Eppendorf tubes. A volume of 100 μL of each supernatant sample was transferred to a 96-well plate in triplicate. 10% Triton-X (10 μL) was added to each well. The Triton-X was added to all samples to ensure comparability since it was observed to slightly increase the fluorescence of rhodamine B. The maximum release was determined by the addition of 10% Triton-X (10 μL) to a non-illuminated, fridge-preserved sample, and the background fluorescence was determined by analyzing the supernatant of ultracentrifuged samples kept at +4 °C temperature. The fluorescence of released rhodamine B dextran was measured by a Varioskan Lux plate reader at excitation and emission wavelengths of 565 nm and 590 nm respectively. The percentage of rhodamine B dextran released was calculated by using eqn 1.

### Calcein release from PdBu_3_PrOH_2_ liposomes in anaerobic conditions

To study the calcein release in a hypoxic environment, a stock solution of sodium sulfite (1.65 M) was used. Sodium sulfite (Na_2_SO_3_) is a molecular oxygen scavenger that inhibits the reactive oxygen species generation by depleting ground-state oxygen. The liposomes loaded with 0.3 M% PdBu_3_PrOH_2_ and calcein were prepared and characterized beforehand as previously described. Two purified PdBu_3_PrOH_2_-liposome samples of 250 μL were diluted to 2500 μL of total volume with HEPES buffer (pH 7.4), whereas one of the samples included 20 mM Na_2_SO_3_. The samples were preserved at +4 °C for 24 hours. The pH of HEPES was measured before and after the addition of Na_2_SO_3_ to ensure the continuousness of stable experiment conditions. After 24 hours, the liposome samples were light-activated with 630 nm, 450 mW cm^−2^ laser for 11–266 seconds by using Modulight ML8500 automatic biomedical illumination instrument (Modulight, Inc, Tampere, Finland). A control sample on the same plate was shielded, whereas a cold control sample was kept at +4 °C and shielded from the light. 10% Triton-X (10 μL) was added to a third control sample to determine the maximum release from the PdBu_3_PrOH_2_-loaded liposomes. The fluorescence of released calcein was measured by a Varioskan Lux plate reader by using an excitation wavelength of 493 nm and an emission wavelength of 518 nm. Each study was conducted in triplicate, and the mean release percentages and standard deviations were calculated. The release based on the average of the experiments was determined according to eqn (1).

### DOTAP oxidation in toluene

DOTAP (3.14 g L^−1^) and PdBu_3_PrOH_2_ (23.3 mg L^−1^ or 92 mg L^−1^) were dissolved in toluene and illuminated for 1, 5, 15, 30, and 60 min with 625 nm wavelength, 189 mW cm^−2^ by using with ThorLab LED (USA). After the illumination samples were diluted with methanol in ratio 1 : 75 and oxidation products were analyzed by using LC-HRMS, Agilent Technologies (USA).

### HPLC-HRMS analysis

Briefly, lipids were extracted with a methanol : chloroform 1 : 2 (v/v) solution from 1 mL of illuminated and non-illuminated liposome samples. The organic layer was collected, evaporated, and resuspended again in chloroform.^23^ Samples in chloroform were diluted with methanol and used for HPLC-MS analysis. LC-HRMS data acquisition was performed using HPLC-ESI-TOF, Agilent 1260 Infinity II LC coupled with AccuTOF 4G LC-plus, Agilent Technologies (USA) in positive electrospray ionization mode. For the chromatographic separation, a Luna C5 reversed phase column (5 μm, 4.6 mm × 50 mm, Phenomenex) with a C5 reversed phase guard cartridge were used. Mobile phase consisted of 95 : 5 water : methanol (v/v) for phase A, and 60 : 35 : 5 propan-2-ol : methanol : water for phase B. 0.1% (v/v) formic acid and 5 mM ammonium formate were added to each mobile phase. The gradient started after 5 min at 0% B and then increased to 100% B over 20 min, followed by 100% B for 15 min, before equilibration for 5 min at 0% B. The flow rate was 0.2 mL min^−1^. An electrospray ionization (ESI) source was used. Capillary voltages were set to 2000 V. Drying gas temperature was 250 °C. Data was collected using an *m*/*z* range of 50–1000 in extended dynamic range. For targeted analysis, the corresponding *m*/*z* for each ion was extracted. DOTAP: *m*/*z* 662.60 (M)^+^; Epoxide/LOOH: *m*/*z* 694.59 (M)^+^; DiLOOH: *m*/*z* 726.59 (M)^+^; Vic-Alcohol: *m*/*z* 696.61 (M)^+^; dialdehyde *m*/*z* 442.32(M)^+^ (see ESI,† Fig. S1) Peaks of DOTAP and its oxidation products extracted from liposomal membrane were presented as molecules of lipids per 1000 molecules of DSPE-PEG, since DSPE-PEG was not oxidized, and peak area of DSPE-PEG remained relatively constant. The peak areas of DOTAP and its oxidation products obtained from illuminated samples in toluene were manually integrated and were presented as ion counts.

### Molecular dynamics simulations of lipid oxidation in liposomal membrane

The GROMACS simulations software version of 2022 was used in carrying the molecular dynamics simulations.^35^ The CHARMM36m forcefield were used for all lipid molecules^36–38^ and the TIP3P model was used for water.^39^ Lipid aldehyde parameters were taken from Wiczew *et al.* (2021)^40^ whereas as hydroperoxides parameters were generated with the CHARMM-GUI automated parametrization tool.^41^ The corresponding aldehyde and hydroperoxide parameters were used to generate oxidated DOTAP lipids with both acyl chains oxidated. Three different simulation systems were constructed as shown in the Table 2. 30% of phospholipids were oxidated to aldehydes or hydroperoxides in aldehyde and DiLOOH systems, respectively.

**Table 2:** Simulated systems and their molecular compositions

	DOTAP	DSPE-PEG	Oxidized lipids	Cholesterol	Water
DOTAP	90	10	0	100	18 832
Aldehyde	60	10	30	100	18 832
DiLOOH	60	10	30	100	18 832

The energy minimization process involved utilizing the steepest descent algorithm with 5000 minimization steps for system optimization prior to commencing simulations. Initially, lipids underwent a simulation lasting up to 1 nanosecond, during which position restraints were applied to the lipid head group and tail carbon atoms in the *Z* direction. The force constant for these restraints was set at 1000 kJ mol^−1^ nm^−2^ to prevent artificial separation of the lipid monolayers due to badly placed atoms. Subsequently, the position restraints were removed, and all systems underwent simulations lasting up to one microsecond. An isothermal–isobaric ensemble with constant NPT was utilized for the simulations. Proper pressure was maintained win the system with the Parrinello–Rahman barostat and coupling constant of 5 ps^−1^.^42^ Temperature was set to 310 K and handled with Nose–Hoover thermostat and coupling constant of 1.0 ps.^43^ Lipids and water (with ions) were coupled separately to heat baths. To manage the electrostatic interactions, we utilized the Particle-Mesh Ewald (PME) summation approach, incorporating a real-space cut-off of 1.2 nm.^44^ The Lennard-Jones interaction cut-off was established at 1.2 nm, with the implementation of a force-switch van der Waals (vdw) modifier initiated at 1.0 nm. Constraints on bonds involving hydrogen were enforced using the LINCS algorithm, and a time step of 0.002 ps was employed.^45^ The last 900 ns were used for analysis. Density profiles and water permeation analysis were conducted with gmx density and gmx select programs, respectively. The order parameters were calculated with the Membrainy suite.^46^ The visual molecular dynamics (VMD) program was utilized to render the figures.^47^

## Data availability

The data supporting this article have been included as part of the ESI.†

## Author contributions

O. L.: methodology, formal analysis, investigation, writing – original draft, visualization. R. K.: methodology, investigation, visualization. A. K.: investigation, writing – original draft, visualization, formal analysis T. L., N. D., A. E.: conceptualization, supervision, funding acquisition, writing – review & editing.

## Conflicts of interest

There are no conflicts to declare.

## Supplementary Material

MA-005-D4MA00535J-s001
