# Supplementary material for: The role of lipid oxidation pathway in reactive oxygen species-mediated cargo release from liposomes
Source: Mater Adv. 2024 Oct 12;5(22):8878–88. doi: 10.1039/d4ma00535j (PMC11491990; doi:10.1039/d4ma00535j)
Supplement: MA-005-D4MA00535J-s001 [file MA-005-D4MA00535J-s001.pdf]

Supplementary Information (SI) for Materials Advances.
This journal is © The Royal Society of Chemistry 2024

Supporting Information

The role of lipid oxidation pathway in reactive oxygen species-mediated cargo release from liposomes

Olga Lem,[a] *Roosa Kekki,*[b] *Artturi Koivuniemi,*[b] *Alexander Efimov,**[,a], *Nikita Durandin,**[, a] *Timo Laaksonen**[, a,b]

[a] Tampere University, Engineering and Natural Science, Materials Science and Environmental Engineering, Tampere, Finland

[b] University of Helsinki, Faculty of Pharmacy, Division of Pharmaceutical Biosciences, Helsinki, Finland

The loading efficiency:

The loading efficiency based on the average of the experiments was defined as Equation 1. states (Equation 1.). The actual of photosensitizer encapsulated in the liposomes was determined by the addition of 10 mL of 10 % Triton-X. Based on the absorption values of the photosensitizer the mass was determined. The total mass is the mass of the photosensitizer and lipids in the liposomes.

$$LE\% = \frac{M_{actual}}{M_{total}} x\ 100$$

Equation 1. The equation for defining of the loading efficiency. The LE% is the loading efficiency, M_{actual} is the actual amount of photosensitizer encapsulated in the liposomes, M_{total} is the total mass of the photosensitizer and lipids in liposomes.

The encapsulation efficiency:

The encapsulation efficiency based on the average of the experiments was defined as Equation 1. states (Equation 2.). The maximum release of calcein and Rhodamine B dextrans from the liposomes was determined by the addition of 10 μL of 10 % Triton-X. The quantitative fluorescence of the compounds was measured by a Varioskan Lux plate reader. The concentrations of the released compounds were received by placing the measured values of fluorescence to calibration curves.

$$EE\% = \frac{C_{actual} \times DF}{C_{theoretical}} x\, 100$$

Equation 2. The equation for defining of the encapsulation efficiency. The EE is the encapsulation efficiency, C_{actual} is the maximum concentration measured to be encapsulated in the liposomes, $C_{theoretical}$ is the original concentration of the hydration solution, and DF is the dilution factor used to dilute liposome solution.

C_{actual} is received from calibration curves.

Calcein: C hydration was 30 mM

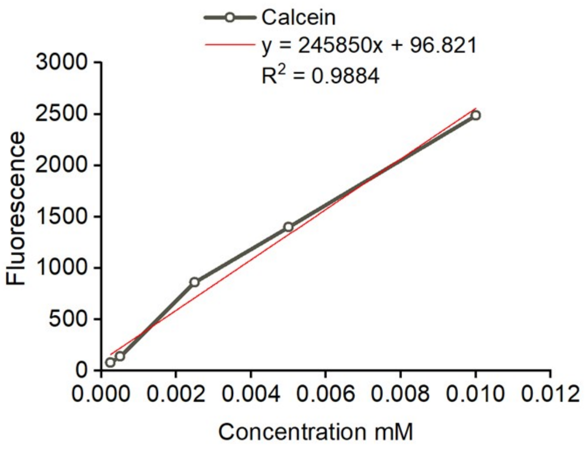

Dextrans: C hydration is 1 mg/ml

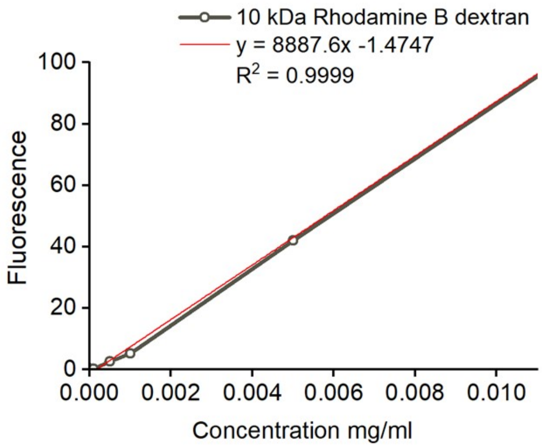

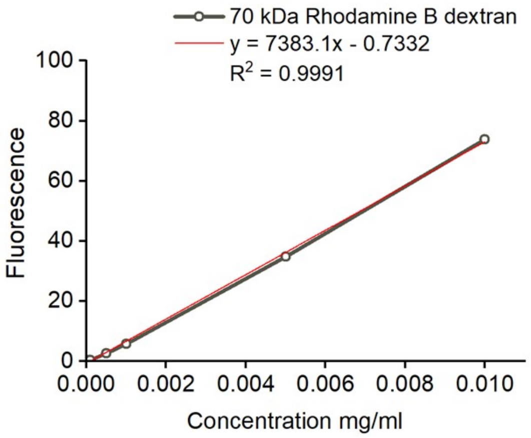

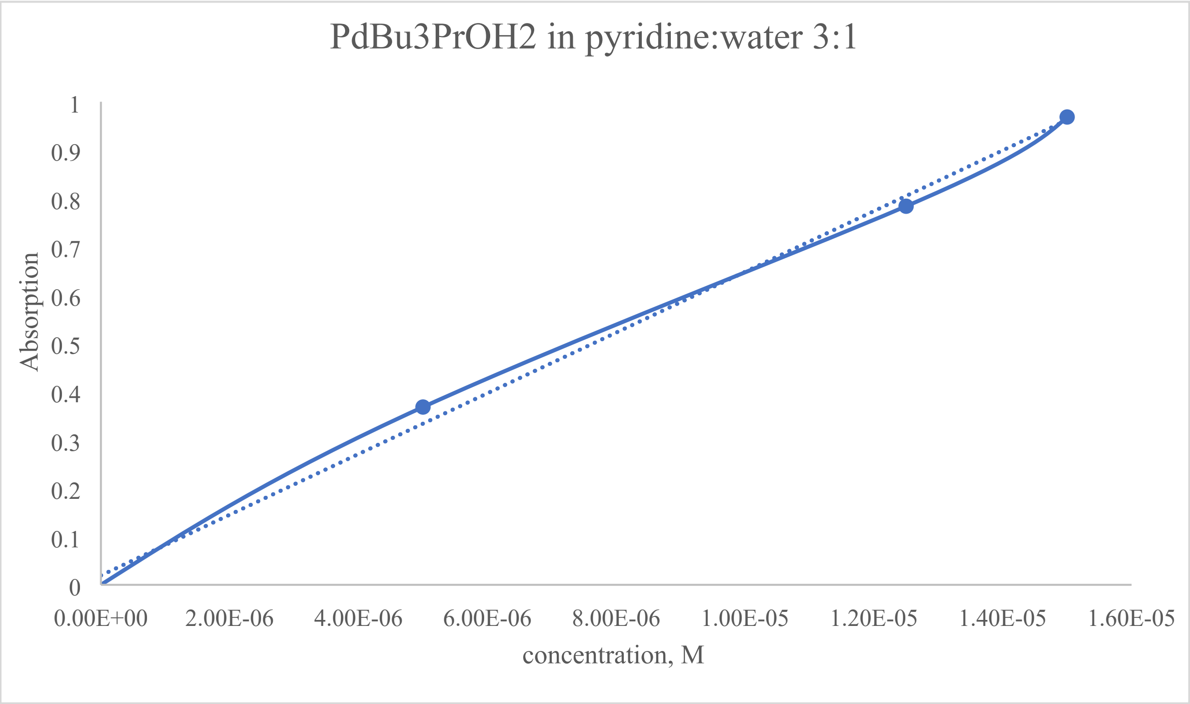

Table S1. Loading efficiency and encapsulation efficiency of $PdBu_3PrOH_2$

	0.3 M%	1 M%	2 M%
Loading efficiency, %	0.38	0.46	0.76
Encapsulation efficiency, %	88.9	31.7	26.4

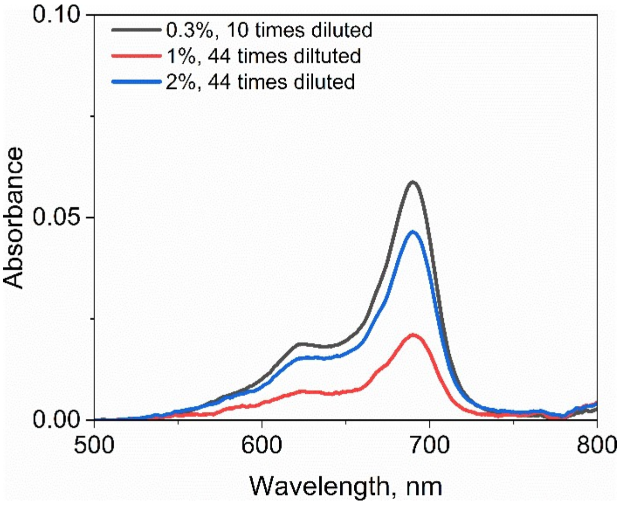

Figure S4. Absorbance of $PdBu_3ProH_2$ pyridine: water (3:1) ratio. Dilution of each measured sample is presented in the legend of the graph.

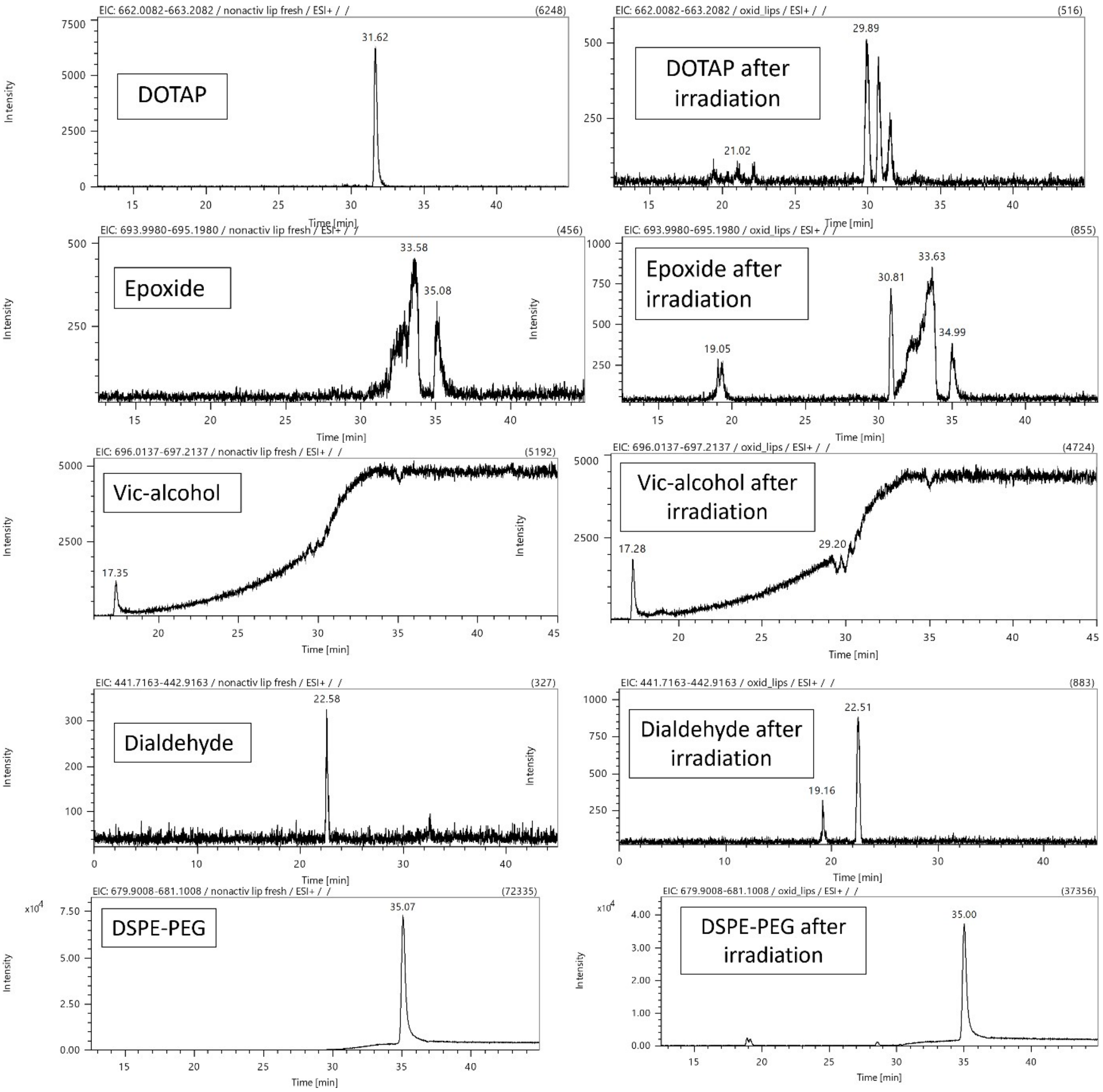

Figure S1. Ion chromatograms of DOTAP and its oxidized products before and after irradiation.

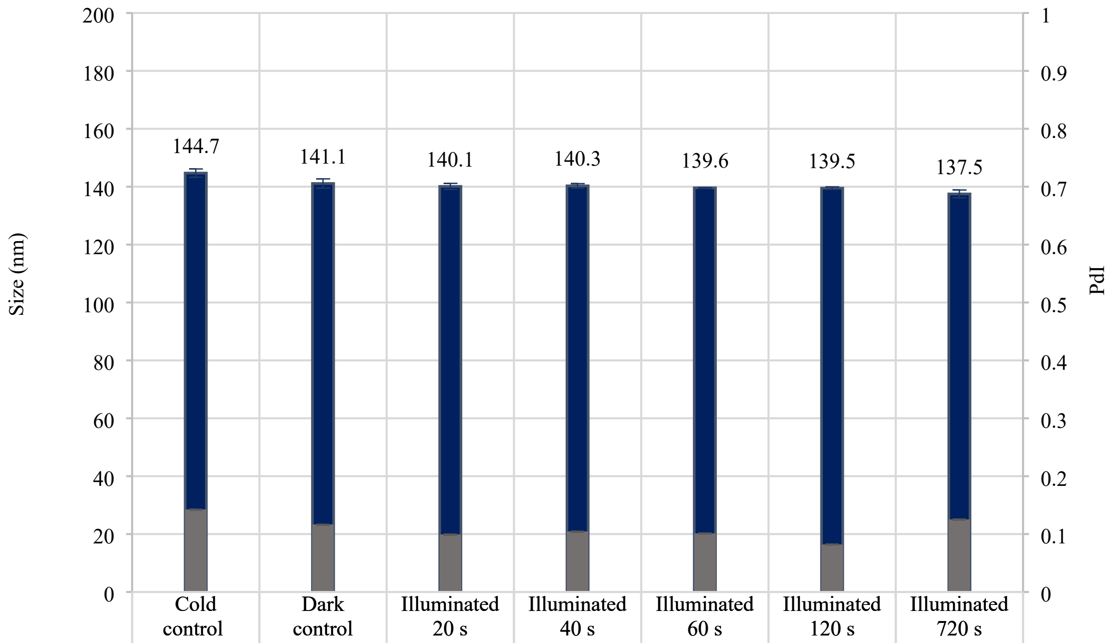

	Cold control	Dark control	Illuminated 20 s	Illuminated 40 s	Illuminated 60 s	Illuminated 120 s	Illuminated 720 s
Size average (nm)	144.7	141.1	140.1	140.3	139.6	139.5	137.5
PdI	0.142	0.116	0.099	0.104	0.101	0.082	0.125

Figure S2. Size shrinking analysis for calcein loaded 2M% $PdBu_3PrOH_2$ liposomes.

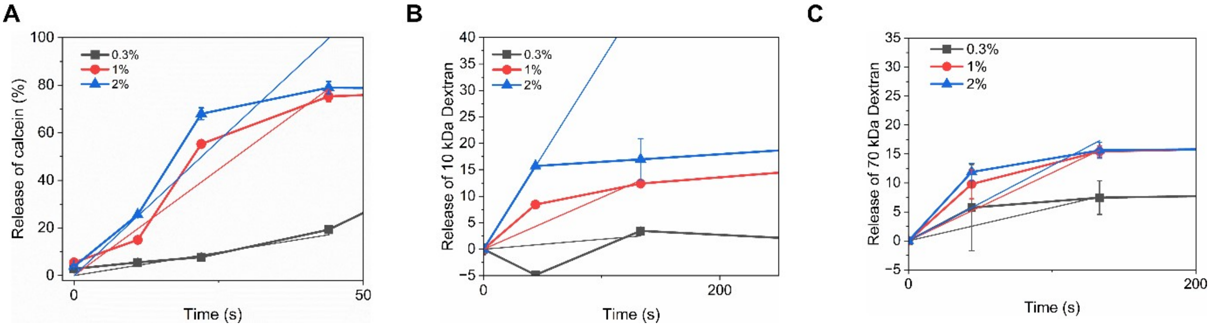

Figure S3. Linear fits of the release studies: A) release of calcein B) Release of 10 kDa, C) release of 70 kDa.

Table S2. Rate constants of cargo release from liposomes loaded with different amounts of $PdBu_3PrOH_2$.

	**10 kDa**	**70 kDa**	**calcein**
**0.3 M%**	0.018	0.057	0.389
**1 M%**	0.097	0.117	1.784
**2 M%**	0.355	0.130	2.265
